# Supplementary material for: STAT3 Mutation Is Associated with STAT3 Activation in CD30+ ALK− ALCL
Source: Cancers (Basel). 2020 Mar 16;12(3):702. doi: 10.3390/cancers12030702 (PMC7140109; doi:10.3390/cancers12030702)
Supplement: Supplementary file 1 [file cancers-12-00702-s001.pdf]

Supplementary Materials: *STAT3* Mutation Is Associated with STAT3 Activation in CD30⁺ ALK⁻ ALCL

Emma I. Andersson, Oscar Brück, Till Braun, Susanna Mannisto, Leena Saikko, Sonja Lagström, Pekka Ellonen, Sirpa Leppä, Marco Herling, Panu E. Kovanen and Satu Mustjoki

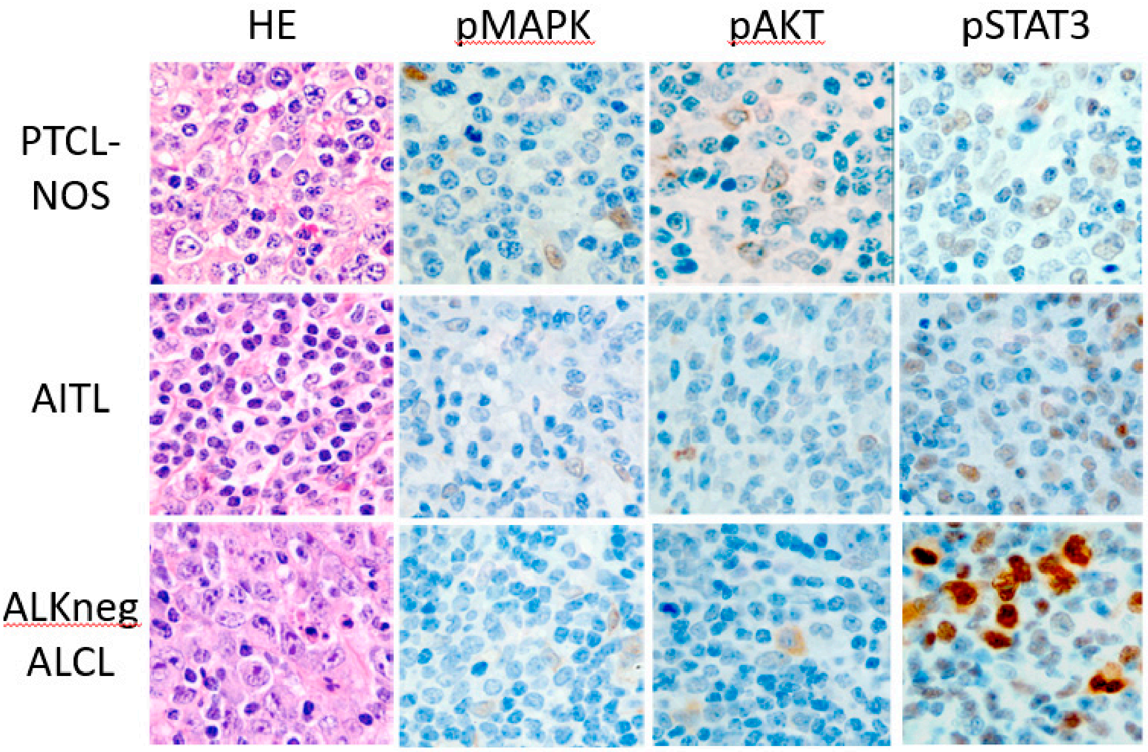

Figure S1. pMAPK, pAKT and pSTAT3 staining of peripheral T-cell lymphomas (PTCLs); not otherwise specified (NOS), angioimmunoblastic T-cell lymphoma (AITL) and ALK negative anaplastic large cell lymphoma (ALCL).

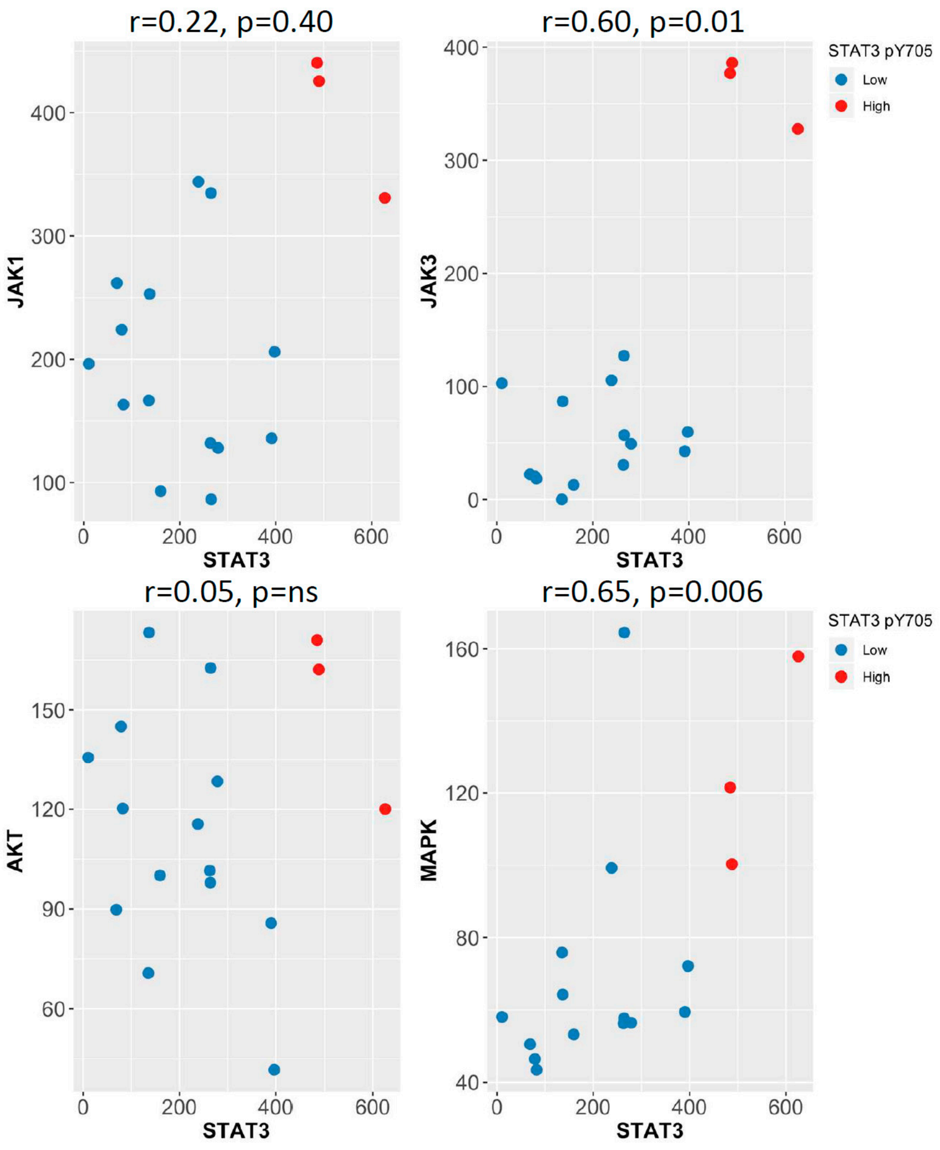

Figure S2. *STAT3* expression was correlated (Spearman correlation) with *JAK1*, *JAK3*, *AKT* (arithmetic mean of *AKT1* and *AKT3*) and *MAPK* (arithmetic mean of *MAPK6*, *MAPK11*, *MAPK12* and *MAPK14*) expression (CPM) in T-cell neoplasia cell lines from the CCLE dataset. Cell lines are labelled based on STAT3 phosphorylation defined with RPPA.

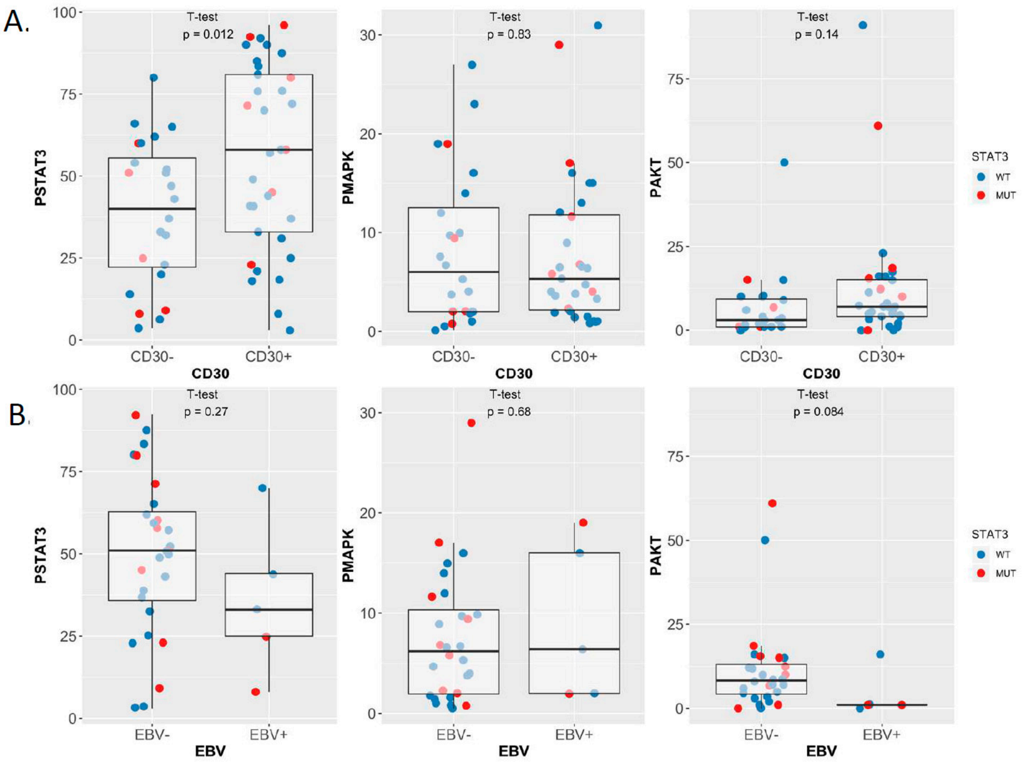

Figure S3. (A) Comparison between pSTAT3, pMAPK and pAKT expression and tumor CD30 and (B) EBV phenotype with unpaired t-test.

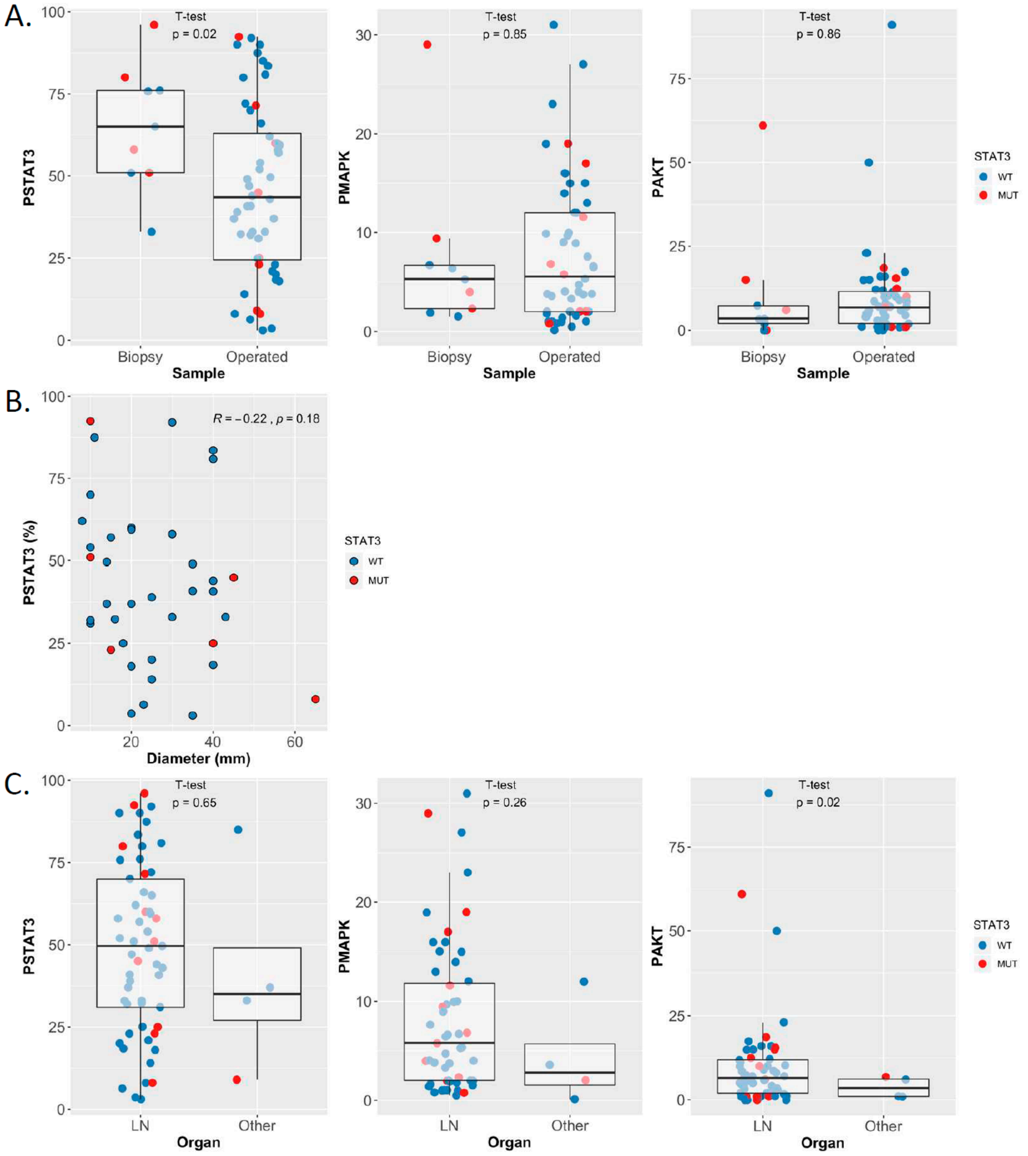

Figure S4. (**A**) Comparison between pSTAT3, pMAPK and pAKT expression and sample type. (**B**) Pearson correlation between pSTAT3 expression and sample diameter. (**C**) Comparison between pSTAT3, pMAPK and pAKT expression and organ origin of the sample. Unpaired T-test has been used for comparison.

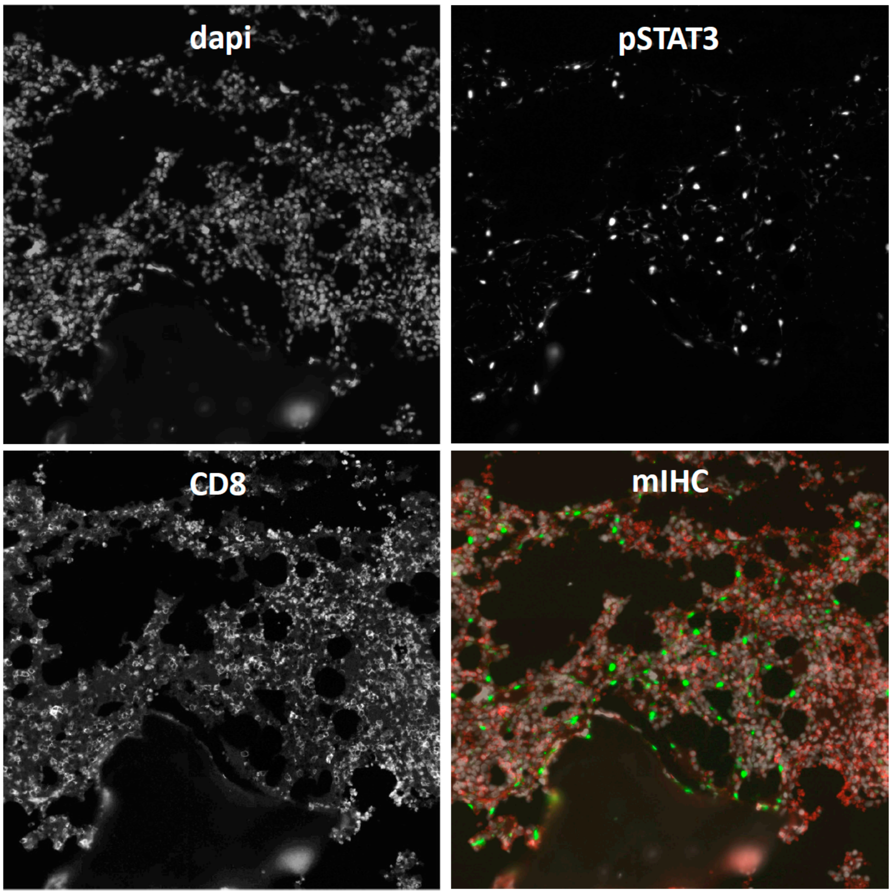

Figure S5. Multiplex immunohistochemistry (mIHC) staining of pSTAT3^{Tyr705} in CD8^{+} large granular lymphocyte (LGL) leukemia as a positive control stain for pSTAT3 antibody. PSTAT3 and CD8 were stained and registered in Cy3 and Cy5 channels, respectively. Dapi was used as nuclear counterstain. The first three images represent single stainings and the last figure their composite image.

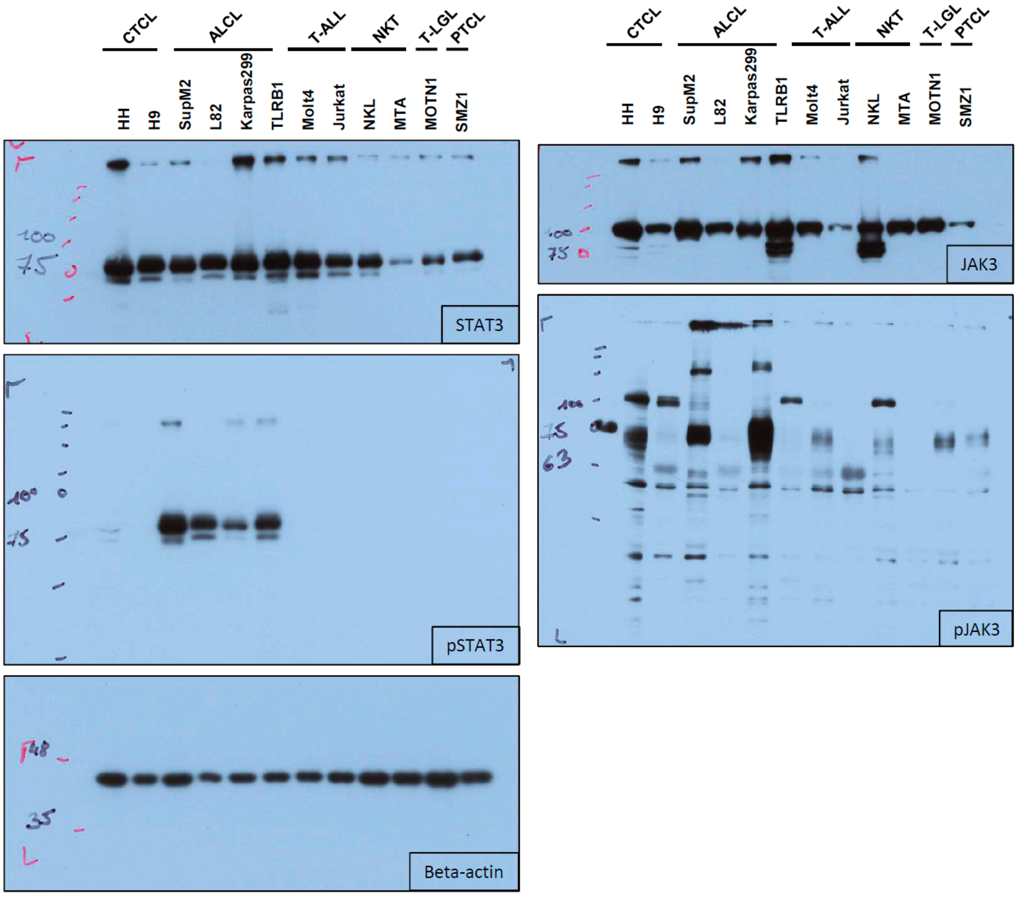

Figure S6. Full western blot of p-STAT3 (Tyr705), STAT3, p-JAK3 (Tyr980/981), and JAK3 in T cell malignancy cell lines. Beta-actin is used as a reference protein. Molecular weights are annotated in the left for each corresponding protein as the staining was digitalized with colorimetric detection.

Table S1. Cell lines and *JAK/STAT* mutation status.

**Disease**	**Cell lines**	**Phenotype**	**JAK/STAT mutation status**
CTCL	HH	CTCL, representing aggressive leukemic CD30+ Mycosis Fungoides and/or ALCL	no mutations
	H9	CTCL	missense JAK1/3
ALCL	SUP-M2	ALCL, ALK+	no mutations
	L82	ALCL, ALK+	no mutations
	Karpas 299	ALCL cell line (of T-cell type) derived from a lymphoma termed at the time “histiocytic high grade	no mutations
	TLBR1	ALCL, ALK-	missense STAT3 (S614R)
T-ALL	MOLT4	T-ALL	nonsense JAK1<br>missense STAT3 (A406V)
	Jurkat	Immature TCRαβ + T-cell line (type T-III cortical) derived from relapsed childhood T-ALL	nonsense/missense STAT5B (T724M, L165L, L142P and Q368fs)<br>missense STAT3 (R729fs and L78F)
NKT	NKL	NKT	no mutations
	MTA	NKT	no mutations
T-LGL	MOTN-1	T-LGL	missense JAK1
PTCL	SMZ-1	PTCL-NOS	no mutations

Table S2. Clinical and pathological characteristics of PTCL patients.

Dg	T %	P-Stat3%	P-MAPK %	P-AKT%	P-Stat5%	Organ	Sample type	Sample size (mm)	CD 3	CD 4	CD 5	CD 7	CD 8	CD 21	CD 30	EB V	MIB %	Gend er	Ag e	Sta ge	LD	ECO G	CH OP	HDC T	Relapse/progre ssion	Deat h	OS time
AITL	70	52	14	3	1	LN	Oper		1	1			0	1	0	0	60	F	59	3	496	3	1	0	0	1	572
AITL	80	33	27	3	2	LN	Oper	30	1	0	1		0	1	0		50	M	63	4	350	3	1	1	1	1	528
AITL	65	57	12	8	9	LN	Oper	15	1	1	1		0	1	1	0	55	F	51	4	634	4	1	1	0	0	2185
AITL	75	66	4	10	10	LN	Oper		1	1	1		0	1	0			F	85	3	238	4	0	0	0	1	26
AITL	85	54	19	2	15	LN	Oper	10	1	1	1		0		0		50	M	57	4	526		1	1	1	1	1717
AITL	55	31	15	91	7	LN	Oper	10	1	1	1	1	0	1	1		40	M	69	3	245	2	1	0	1	1	1115
AITL	40	25	2	1	0	LN	Oper	40	1	1	1		0	1	0	1		M	68	4	530	3	0	0	0	0	49
AITL	35	32	10	1	1	LN	Oper	10	1	1					0			M	80	3	534	2	0	0	1	1	218
AITL	75	47	23	9	3	LN	Oper		1	1	1	0	0	1	0		60	M	61	4	406	2	1	0	1	0	218
AITL	60	23	16	15	10	LN	Oper		1	1	1		0	1	0	0	40	F	63	4	235	1	1	1	1	1	2621
AITL	80	21	13	5	5	LN	Oper		1	1	1	1	0	1	1		70	F	77	3	272	2	1	0	1	1	2601
AITL	75	49	15	16	2	LN	Oper	35	1	1	1	1	0	1	1	0	70	M	74	3	254	1	1	0	0	1	259
AITL	70	43	1	1	2	LN	Oper		1	1	1	1	0	1	0	0	30	M	78	3	360	1	0	0	0	1	308
AITL	70	25	1	5	4	LN	Oper	18	1	1	1	0	0	1	1	0	60	M	64	4	263	1	1	1	1	1	445
AITL	90	58	31	23	0	LN	Oper	30	1	1	1	0	0	1	1		70	M	84	3	266	3	0	0	0	1	79
AITL	40	45	17	10	4	LN	Oper	45	1	1	1	0	0	1	1	0	90	M	57	3	237	2	1	0	1	1	698
AITL	70	44	16	16	1	LN	Oper	40	1	1	1	0	0	1	1	1	30	M	67	3	210	1	1	0	1	1	4739
AITL	60	60	1	1	1	LN	Oper	20	1	1	1	0	0	1	0	0	50	F	86	3	355	1	0	0		1	62
AITL	75					LN	Oper	35	1	1	1		0		0		40	M	31	3	372	0	0	1	1	1	1238
AITL	50	33	12	6	2	Orophar ynx	Oper		1	1	1		0	1	0			F	74	1	194	1	1	0	1	1	2339
AITL	95	60	4	1	1	LN	Oper	20	1	1	1	1	1		0		85	F	63	3	386	2	1	1	1	1	1466
AITL	60	65	5	4	1	LN	Biopsy		1	1	1	1	0	1	0	0	30	F	68	3	229	1	1	0	0	1	125
AITL	60	51	9	15	1	LN	Biopsy	10		1	1	1	0	1	0	0	50	M	69	3	326	1	0	0		1	282
AITL	60	62	2	10	1	LN	Oper	8	1	1	1		0	0	0	0	40	F	45	4	448	1	1	1	0	0	2284
AITL	60	51	7	2	1	LN	Biopsy		1	1	1	1	0	1	0	0	60	F	79	2	212	0	0	0	1	1	392
AITL		18.4	3.3	5.4		LN	Oper	40	1	1	1	1	0		1			M	67	2	397	1	1	1	0	0	909
AITL		32.3	8.9	5.9		LN	Oper	16	1	1	1	0	0	1		0	35	F	78	4	303	2	1	0	1	0	1322
AITL		39.0	9.9	8.5		LN	Oper	25	1	1	1	0	1	0		0	70	F	67	3	353	3	1	0	1	1	260
AITL		49.6	3.8	8.6		LN	Oper	14	1	1	1	1	1	1		0	50	M	66	4	265	1	1	1	0	1	603
AITL		40.9	3.8	4.1		LN	Oper	35	1	1	1	1		1	1			M	40	4	241	1	1	1	0	0	2059
ALCL ALK-	90	3	4	7	1	LN	Oper	35	0	1	0		0		1	0	90	M	70	3	268	1	1	0		1	1254
ALCL ALK-	96	8	1	2	0	LN	Oper		1	0	0		0		1		90	F	46	2	178	0	1	0	1	0	4743
ALCL ALK-	90	96	4	6	1	LN	Biopsy		0	0	0	0	0		1		90	F	40	4	229 0	2	1	1	0	0	3237
ALCL ALK-	90	90	1	11	4	LN	Oper			1	1	0	0		1		90	F	84	4	169	4	0	0		1	54
ALCL ALK-	90	80	10	50	28	LN	Oper		0	1		0			0	0	95	F	73	4	175 8	3	1	0	1	1	298
ALCL ALK-	50	92	1	0	69	LN	Oper	30	0	1	0	0	0		1		60	F	44	2	212	1	1	1	0	0	1982
ALCL ALK-	60	58	29	61	6	LN	Biopsy		0	1	0	0	0		1	0		M	60	2	364		0	0	1	1	2008
ALCL ALK-	85	72	7		9	LN	Oper		0	1	1		0		1		90	M	59	4		4	0	0		1	13
ALCL ALK-		83.5	1.4	4.5		LN	Oper	40	0	1		0	0	0	1	0		F	50	4	279	1	1	0	0	0	1519
ALCL ALK-		23.0	6.8	18.6		LN	Oper	15	1	1	1	1	0		1	0	90	M	86	N/D	N/ D	3	0	0		1	245
ALCL ALK-		71.5	11.6	15.5		LN	Oper		0	0	0	0	0		1	0	75	M	66	4	366	2	1	0	0	1	75
ALCL ALK-		92.4	5.8	12.4		LN	Oper	10	0	1	0	1	0		1	0	75	M	53	4	136	1	1	1	1	1	696
ALCL ALK-		40.8	3,9,	14.9		LN	Oper	40	1	1	1	1	0		1		90	M	49	4	774		1	0	0	0	1225
ALCL ALK+	90	85	4	1	1	Orophar ynx	Oper		0	1	0		1		1			F	30	4	187	1	1	0	0	0	2166
ALCL ALK+	20					LN	Oper		0	1			0		1		90	M	29	4	172	4	1	0	0	0	2059

ALCL ALK+	75	90	5	4	7	LN	Oper		0	1	0	0	0		1		80	F	42	3	186	1	1	0	0	0	3694
ALCL ALK+	90	80	2	0	0	LN	Biopsy		0	1	1		1		1	0	80	F	13	3	151	0	0	0	0	0	2002
ALCL ALK+	90	70	2	1	0	LN	Oper	10	0	1	1	1	1		1	1	75	M	8	4						1	97
ALCL ALK+		75.8	1.9	3.3		LN	Biopsy		0	1	1	0	0		1		80	F	71	3	191	1	1	0	0	0	1479
ALCL ALK+		80.9		17.4		LN	Oper	40	0	1	0	1	0		1		70	F	31	3	168	1	1	0	0	0	1594
ALCL ALK+		76.0	1.5	7.3		LN	Biopsy		0	1	0	0	0		1		70	M	67	2	152	0	1	0		0	1526
PTCL NOS	90	8	19	1	1	LN	Oper	65	1	1	1		0		0	1	70	M	70	4	468	3	1	0	1	1	213
PTCL NOS	85	4	1	0	0	LN	Oper	20	1	1	1		0	0	0	0		M	47	4	673	1	1	1	1	0	819
PTCL NOS	80	14	2	4	5	LN	Oper	25	1	1	1		0	0	0			F	79	2	248	1	0	0	0	1	4199
PTCL NOS	70	37	0	1	0	Pancreas	Oper	14	1	1	1		0	0	0			F	52	2	265	1	1	1	0	0	5193
PTCL NOS	75	18	9	7	3	LN	Oper	20	1	1	1		0		1			M	47	3	388	0	1	1	0	1	197
PTCL NOS	30	9	2	7	10	Oropharynx	Oper		1	1	1	1	1		0	0	50	M	41	2	220	1	1	1	0	0	3655
PTCL NOS	80	37	5	7	2	LN	Oper	20	1	1	1	1	0	1	1	0	80	F	51	4	404	3	1	1	0	0	3196
PTCL NOS	80	33	6	0	5	LN	Biopsy	43	1	1	1	1	0	0	1	1	70	F	80	4	435	0	0	0	1	1	538
PTCL NOS		87.4	6.6	12.1		LN	Oper	11	1	1	1	1	0	1	1	0	70	M	47	3	333	1	1	1	1	0	1729
PTCL NOS		6.3	7.6	1.6		LN	Oper	23	1	1	1	1	0		0		70	M	64	4	3200	3	1	1	1	1	378
PTCL NOS		59.4	1.6	11.8		LN	Oper	20	1	1	1	1	1			0		M	42	4	349	3	1	1	0	1	207
PTCL NOS		20.0	2.0	10.3		LN	Oper	25	1	1	1	1	0		0		60	M	79	1	121	2	0	0		1	1407

Abbreviations. Diagnosis (Dg). Tumor proportion (T%). Lymph node (LN). Lactate dehydrogenase from blood (LD). Overall survival time (OS time).

Table 3. Locus-specific primers for *STAT3* and *STAT5B* exons and RHOA G17V mutation hotspot.

Site	Forward primer	Reverse primer
STAT3 exon 2	TCCCCATCACCTGTACCCAT	TGACACCTGTGTTGGGCAAT
STAT3 exon 3	ACACTAACACCCGACTCTGC	TGTATGCGTCGGCTTCAGAG
STAT3 exon 4	TCCATTCCTCCCAGACCAGG	GCTCTGAAGCCTTTGTTCCG
STAT3 exon 5	CCGAGGCTTGTAACTTGCAT	TTCCCTTCCTCTTGTGATGG
STAT3 exon 6	GACCAGGCTCCTTTGAGGAC	CTCTTGGGGATACTGCCTGC
STAT3 exon 7	CCGATCTAGGCAGATGTTGG	TTCCCTCAGGTCAAGGAGTTT
STAT3 exon 8	CTGTGGGCCTGCAGTTAAGA	GTTCCTGCTCTGGAGTTGACT
STAT3 exon 9	AAGAGAAGATGGGCTCACGC	TCCCCTTCTCCATCTCACCT
STAT3 exon 10	TGGAAAGAATGACCCTGGCC	CACGTGGTAGAGTGAGAGGC
STAT3 exon 11	AATGCACCCCAAGGCTTTTG	CCTCCCACAGTGCTGAGATT
STAT3 exon 12-14	CAAGGAAAACACCCCAGTTG	AAATAACAGGTGGTCAAAGTAGGC
STAT3 exon 15	ATTGCCAGATGGGATGCCAA	CCACACCTGGCCTAAGAGTG
STAT3 exon 16	GAGGAGAACTGCCAGCTCAG	CCTTTCATTCTGAGCCCCGT
STAT3 exon 17	AGGGAGAAGGGGTGAAATGC	TGCCCCCTCCTTTTAGTTGG
STAT3 exon 18	CCTTGCCAGCCATGTTTTCC	AACCTCTTGACCCCAAGCTG
STAT3 exon 19	GTGCACACTCTGTCCAACCT	GCTTGAAGGCCCTGAACTCT
STAT3 exon 20	GGAGTCAAGGCCATCTCCAC	TGGATGCCCTGTTAGCAATA
STAT3 exon 21	CCCAAAAATTAAATGCCAGGA	GGTTCCATGATCTTTCCTTCC
STAT3 exon 22	CTCACCCAGTGTCCCATTCC	GGCAGATGGAGCTTTCCAGA
STAT3 exon 23	GACCAGCTCTCGGTGTGTAC	TGGAGACCAGAGTTTGATGGC
STAT3 exon 24	GGCACTTGTCTAAGAACAACAACA	AGTTGCAGAGGGTGGACAAC
STAT5B exons 14-15	CTGACCTCAACAAATAGTAAGTACCC	TTCCAAGTGTTACTCTGGTGTTC
STAT5B exon16	TGTTGGGGTTTTAAGATTTCC	CAAATCAGAATGCGAACATTG
STAT5B exon 17	CCCAGGGCTGAGACAGTTT	AGATTGCACCACCGTACTCC
STAT5B exon 18	TGGATTCCTTTGACCCCAGC	AGATACCCCTTGGTCCCCTC
STAT5B exon 19	CTGGTGGCCTGTGGGGCTTG	TCTGTCTGTGGCCCCTCTGCT
RHOA G17V	TGGAAAATGGCATCAGTTGTTATG	GCATTGCAGGTAATATCTGTGTTT
JAK1 exon 14	CCACCCACCCCTTTGAAAGA	ATTGATGTTCAGGGCCTGGG
JAK1 exon 20	CCACCAGCTAGCATGTCAGA	CTGTGTGTTCCGTGGCCTA
JAK1 exon 24	ACCAGGCACACCTTTGTTCA	AGGGGATGAAGGAGAGGACC
JAK3 exon 11	TCTCTGGACCCCAGACTGAG	GTCTCAACAGCAGCAGCAAC

Table 4. Densitometry readings of western blot stainings.

	**ß-Actin**	**JAK3**	**STAT3**	**pJAK3**	**pSTAT3**
**HH**	14546719	17062962	17023719	6057477	355163
**H9**	9331062	9270477	15436477	4239406	53192
**SupM2**	13836376	17276012	13283305	1165577	18000619
**L82**	6454355	11489012	13808598	106778	13158012
**Karpas299**	8757012	13082891	17198012	680749	6562891
**TLRB1**	8803426	17857841	16716062	2462820	12840083
**Molt4**	9240719	12835719	15543184	54657	0
**Jurkat**	11107305	3284891	11095184	91778	0
**NKL**	15404719	14683305	9410012	3615941	0
**MTA**	15076891	14215719	2003355	96485	0
**MOTN1**	19614669	14053426	5048305	37243	0
**SMZ1**	12446477	3447891	7313962	66071	0

© 2020 by the authors. Licensee MDPI, Basel, Switzerland. This article is an open access article distributed under the terms and conditions of the Creative Commons Attribution (CC BY) license (http://creativecommons.org/licenses/by/4.0/).
